# ‘One of the greatest injustices of our time’: The impact of social representations of modern slavery in the UK—A mixed methods approach

**DOI:** 10.1111/bjso.12824

**Published:** 2024-12-02

**Authors:** Melanie Haughton, Katia C. Vione, Zoe Hughes

**Affiliations:** ^1^ University of Derby Derby UK

**Keywords:** content analysis, critical discourse analysis, illegal immigration, modern slavery, social representations

## Abstract

This study aimed to examine how social representations of modern slavery and immigration become entangled in newspaper media. 2672 UK newspaper articles were collated from 2013 to 2022 and analysed using Content Analysis (Descendant Hierarchical Classification) and Critical Discourse Analysis (CDA). Two themes and corresponding extracts were identified from the content analysis output and analysed using CDA allowing for the exploration of the role of the hegemonic social representations to understanding how discourses of modern slavery are reproduced through the othering in relation to ethnicity and migration. The two themes that emerged were ‘Leading the way in tackling slavery’ and ‘Making claims.’ The themes combined showed how the media and political discourse positioned the UK government as leading the way in tackling modern slavery. Simultaneously, discourses criminalized migrants and constructed them as the source of social harm, and the obstacles to resolving the social injustices were those opposing anti‐immigration legislation. These two contradictory positions have implications in how modern slavery is understood by the public. More worryingly, it could lead to silence from victims due to the threat of being criminalized.

## INTRODUCTION

Since Theresa May became the UK's Home Secretary in 2010, under David Cameron's premiership (Home Office, 2010), modern slavery has been at the heart of migration policy. With changes in premiership and cabinet ministers, there have been increasingly punitive policies presented, with modern slavery becoming conflated in these discourses. By examining newspaper articles over a 10‐year period, from 2013 to 2022, the emerging discourses around modern slavery and migration show how arguments have become potentially harmful to victims of human trafficking. Although it would be useful to examine the evolution of discourses over the past 10 years, here we will examine how newspapers as media sources have shaped UK's understanding of modern slavery and migration, establishing the need to further explore discourses around modern slavery and migration. This will also highlight the influence of such discourses, and how such discourses need to be considered when policy is developed relating to the issue of modern slavery. By using Moscovici's (2000) theory of Social Representations and Critical Discourses Analysis (CDA); (Fairclough, 2010), we argue how media representations have contributed to the public's understanding of modern slavery and migration. The integration of political discourses put forward by Van Dijk (1993) and Fairclough (2010) will inform the analysis, as well as drawing upon Foucauldian principles of discourse and power. Here we explore how modern slavery has been racialised and in doing so marginalized. We examine how the use of discourses serves to ensure modern slavery continues to go unreported due to the representations that are available in relation to British attitudes towards migrant victims of slavery.

Modern Slavery is a wide‐reaching issue, in terms of who it affects and what it encompasses. According to Anti‐Slavery International (2024), it can be defined as ‘when an individual is exploited by others, for personal or commercial gain. Whether tricked, coerced, or forced, they lose their freedom’. The true extent of modern slavery is difficult to measure. Hulland (2020) highlights the problem, stating that in 2013, it was reported that there were between 10,000 and 13,000 people held in slavery in the UK, but this figure was a massive underestimate. According to the Global Slavery Index (Walk Free, 2024) there are currently around 122,000 individuals being held in slavery in the UK.

The 2014 Immigration Act saw a clamping down on migration and reference to ‘illegal immigrants.’ The following year the Modern Slavery Act 2015 consolidated several laws to tackle the issue of trafficking, forced servitude, and forced labour, as well as ensured that there was special dispensation for victims of modern slavery who were forced to commit crimes. The Act represented a long history of the UK challenging slavery, and was supposed to make it easier to convict traffickers, as well as protect the victims of modern slavery (May, 2016). It is the case that there has been a sharp increase in the number of prosecutions and convictions since the introduction of the Act in 2016. The act has been revolutionary in how governments can prosecute acts of slavery, and has been influential internationally (Shraer, 2019). It is worth noting that 70% of victims of modern slavery identified through the legal system are non‐British citizens. The now scrapped ‘Rwanda Bill’ attempted to undermine the use of the Modern Slavery Act 2015 to prevent trafficking victims being returned to the country from which they were trafficked. Arnell et al. (2023) claimed this was in effect a ban, and politicians in the House of Commons and House of Lords questioned the legality of such clauses (Fraser, 2024). The changes in legislation from the introduction of the ‘Hostile Environment’ to the ‘Rwanda Bill’, have become increasingly draconian, leading victims of slavery unable to report the crimes against them. Such policies racialize and marginalize individuals, and De Noronha (2019) argued there is a clear connection between immigration and race, and immigration controls produce racial meanings and equalities. Arguably, such policies are a response to the needs of the electorate.

Politically, migration has always been a thorny issue since post‐World War II Britian when the UK needed migrants due to skills and labour shortages (Mynott, 2000) and the infamous Rivers of Blood speech in 1968 (Warwick University, 2022). However, not until Tony Blair became Prime Minister has migration become such a crucial part of politics with Jack Straw implementing anti‐migration policy while ignoring the racial undertones of such legislation (Mynott, 2000). Following Labour's election defeat in 2010, Theresa May, as Home Secretary, called for a need to manage migration, drawing on discourses of abusing the system, and statistics of net migration under the Labour government, responsible for the country's woes (Cole, 2019). She focused on community division and tropes of the ‘immigrant family’ and this led to what is often referred to as the ‘hostile environment’. She argued that by removing ‘illegal’ migrants, that social injustices, such as modern slavery would be tackled.

However, attitudes towards migration softened following the 2016 EU Referendum, but over previous years, there has been an increase in opposition to migration from the general public. In 2022, 52% of people stated that they believed that immigration numbers should be reduced and in April 2023, 37% of people felt that the arrival of asylum seekers should be made more difficult, but only 14% wanted this to be the case for Ukrainians (The Migration Observatory, 2023). Research by the Migration Observatory (2023) suggested that there is a racial bias in relation to migration, with non‐EU members, refugees and unskilled workers being identified by participants as problematic migrants. However, it is important to consider how individuals come to these conclusions, and the sources of information in shaping attitudes of people, especially when victims of human trafficking and modern slavery are more likely to be categorized as those identified as problematic in the data collated by the Migration Observatory (2023).

Dando et al. (2016) surveyed a sample of 482 participants in the West Midlands, and found that most participants reported they gained most of their information around modern slavery and trafficking from newspapers, followed by other media sources. The study found that there was a lack of understanding as to what would be considered trafficking and who likely victims were. The participants tended to believe that it was predominantly women who were trafficked, rather than men or children. These findings are unsurprising, as Rodríguez‐López (2018) found that human trafficking is often conflated with prostitution and sexual exploitation, which are arguably overrepresented in the media. The findings showed that there was a focus on the victim to reproduce prostitution narratives of ‘bad prostitutes’ and ‘good victims.’ Furthermore, the focus of such representations in the media was the criminal act rather than the consequences on the victim.

Birks and Gardner (2019) found that local newspapers tended to interchange the terms modern slavery and human trafficking. Modern slavery tended to be used in cases of forced labour and servitude, whereas trafficking was associated with sex trafficking, sexual exploitation, prostitution, and people smuggling. Participants of the focus group in the second part of Birks and Gardner's (2019) research were unaware that exploitation occurred local to them, associating forced labour with countries with poor labour regulations, rather than the UK. For example, participants associated forced labour and servitude with sweatshops in Bangladesh and China. They also associated modern slavery with illegal migration and people smuggling, especially in relation to the recent ‘migration crisis’. There was also the assumption that victims of trafficking were complicit in the act, as it was preferable to the conditions of their home country.

Previous research suggests that the media plays a crucial role in understanding modern slavery and legislation. The impact of media and political discourse have been identified by Van Dijk (Van Dijk, 1993; Van Dijk, 1998; van Dijk, 2015) and Fairclough (1995, 2010). Fairclough (1995) stated that media sources do not simply reflect events, but produce texts based on representations that meet the objectives of those who produce them. Van Dijk (2015) identified how discourses are used for ‘ethnic events’, in that racist discourses serve a social and political ideological purpose, in that they maintain power for the elite. Through the media and political discourse, ‘ethnic events’ like migration or modern slavery enable the use of discourse to maintain the ‘othering’ of racial groups. The ideologies that the representations are based on are an important process in establishing power (Fairclough, 2010).

To understand the process of representations, Moscovici (2000) developed the Social Representations Theory (SRT) and was concerned with locating the ‘social’ in social psychology. Moscovici saw these as fundamental in understanding the psychology of the individual within society. He identified three types of social representations, hegemonic (overarching social representations reproduced by power constructs), emancipatory (produced from the powerless subgroup) and polemic (produced from subgroups due to social conflict). Social representations are produced and reproduced to a point where they can become accepted as ‘general knowledge’ (Fairclough, 2003; Moscovici, 2000). These social representations are not accurately reflections of the lived experiences of individuals to which social representations refer. According to Moscovici (2000) social representations are formed and maintained through processes of objectification and anchoring. Objectification is the process of giving meaning, through imagery and metaphors to abstract concepts, to give a reality to the object. This is followed by ‘anchoring’ whereby the new concrete ‘objects’ become part of the already existing framework of the social group. This is relevant here as it can be used to explain how the different terms for migrants have become conflated in the British media. Augoustinos (2001) used social representations and a discursive approach to understand the implications of political categories on the group and identities.

Based on the existing research, it is apparent that what people know about modern slavery, they learn from the media, predominantly newspapers (Birks & Gardner, 2019; Rodríguez‐López, 2018). It seems that through political discourse and legislation, migration and modern slavery discourses have become conflated putting more people at risk of trafficking (Balch, 2023). Simultaneously there is a growing dissatisfaction in migration for some people in the UK, where attitudes do not reflect actual migration patterns (Foster & Borrett, 2024). This indicates that there is a disparity between actual migration trends and what some believe to be the issue of migration, indicating there is a need to scrutinize sources of information that may be contributing to this misunderstanding. When considering Moscovici's (2000) theory it is possible that the social representations being reproduced in the media contribute to negative attitudes towards migration, specifically modern slavery. Using newspaper articles since 2013, this study aims to understand how social representations of modern slavery and immigration are constructed in newspaper media. First, a content analysis is conducted to take advantage of statistical methods to identify patterns of language and words used in relation to modern slavery. Following this, emerging themes are analysed using CDA, informed by SRT (Moscovici, 2000) to examine the double standards of the media when reporting modern slavery in the UK. Using SRT and CDA, analysis of the newspaper articles will serve to inform on how social representations of modern slavery in the UK is reproduced and the potential consequences in terms of the double standards this creates for them.

## METHOD

### Data sourcing

UK newspaper articles were collated from 2013 to 2022, to account for modern slavery arguments put forward in 2013, and the introduction of the Modern Slavery Act 2015 and the examination of how discourses evolved during the political and legal changes. Nexus Lexus was used to search for national and local newspaper articles. The search used keywords relating to slavery and trafficking: modern, slavery, human, sex, trafficking, county lines, forced servitude, sweat shops. These key terms were added into Nexus Lexus, with inclusion criteria of UK national newspapers. All other news outlets were excluded.

### Inclusion criteria and final sample

**FIGURE 1:**
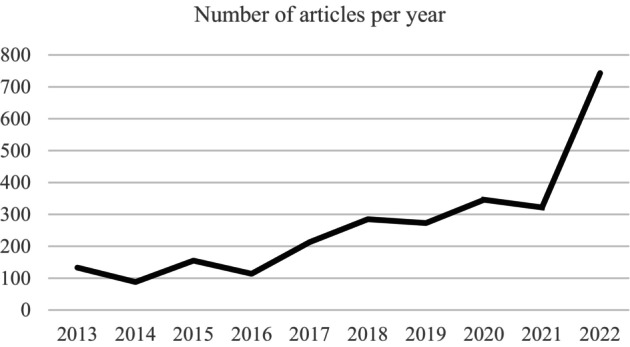
Frequency of articles per year.

Advertisements and repetitions were excluded, alongside articles or TV programmes where slavery or trafficking were not the focus and historic cases of slavery (i.e. inter‐Atlantic slave trade). Exclusion criteria was applied to the article. An initial first reading was conducted removing articles which were advertisements, those making reference to TV programmes, books or other sources of media, and those offering only a historical context of slavery. Articles were also removed if phrases such as ‘it is like modern slavery’ were used to refer to issues that did not fit into the legal definition of modern slavery. Also, articles under 250 words were removed, as these were seen as too limited for qualitative analysis. Once exclusion criteria had been applied, this left a sample of 2672 newspaper articles. Almost three quarters (73%) of the articles were published in the last 5 years, more than a quarter (28%) were published in 2022 alone (see Figure 1).

Articles were published in seventeen different outlets, most were published by The Times, followed by The Independent and The Guardian. The number of articles published by each outlet is shown in Table 1.

**TABLE 1:** Number of articles published by each newspaper.

Newspaper	Number of articles
The Times	487
The Telegraph Online	167
The Guardian	356
The Sun	215
The independent	379
The mirror	108
Daily mail	257
Daily telegraph	231
Sunday times	87
Mail on sunday	27
The observer	2
Daily mirror	122
The independent	191
Sunday telegraph	2
The People	2
The Start Online	24
i	15

### Analysis strategy

Given the large amount of text data, in the first step a content analysis was conducted using the software IRaMuTeq (Interface de R pour les Analyses Multidimensionnelles de Textes et de Questionnaires; Ratinaud, 2009). All articles were organized into a single corpus and analysed through Descendant Hierarchical Classification (DHC), following the Reinert method (Reinert, 1993), a transparent and reproducible method. In this analysis, segments of text (ST) are classified according to their lexicon, based on co‐occurrence and frequency of words, computed by a χ^2^. These segments are then grouped into classes called Elementary Context Units (ECUs), which organize the STs based on vocabulary similarities within each class and differences in vocabulary between classes. The process of grouping similar forms of a word is called lemmatisation. The classes that directly addressed the research question were then analysed using CDA. Due to the volume of the data sets, the content analysis served the purpose of coding and theme generation, so these were based on frequency of themes, and inter‐relatedness. From the data sets, the five highest scoring extracts for each theme were selected and the original article analysed in more detail. This was necessary to ensure that the stages of CDA (Fairclough, 2010) are followed, with the first stage being ‘identifying the social harm’. The advantage of adopting this analytical approach in comparison to other statistical text analyses is that classes are interpreted by the researcher after computation rather than pre‐defined (Montalescot et al., 2024).

### Reflexivity

The aim of this study is to start a wider examination of modern slavery from a psychological perspective. The position we took was that we do not know what we do not know. We, ourselves understood that modern slavery is complex and often misunderstood, including by ourselves. The implication of this is that if people do not know what we mean by modern slavery, how can people report it? Therefore we felt that the first stage should be to understand what commonly held beliefs are. Using newspaper articles from prior to the modern slavery act until relatively recently would give us a sample that would allow for a mixed methods approach. There is always a risk of bias in research, especially with qualitative research, and as a team of two female and one non‐binary researchers, we were aware of our position in society. Initially it was thought that FDA would be sufficient to analyse the data, as this would offer a robust analytic procedure. However, upon reflection, how discourses were used and the assumption of political motivation, meant that critical discourse analysis (CDA) was deemed more suitable. Also, as CDA allows for exploration that is omitted this seemed a more appropriate. Furthermore, Fairclough developed the approach to account for media and political discourse.

### Critical discourse analysis (CDA)

Due to the nature of discourses, CDA accounts for what is present (and what is absent) from mass media, and how it can influence social practices and representations, it is appropriate for this analysis. Due to the potential racialised nature of media representations, this is useful to examine when racial categories are (and not) used. CDA accounts for the impact of discourse and media on the individual, which means it has the potential to interpret not only the newspaper articles, but also their potential impact on group members. This critical approach is crucial for social justice to understand the impact of power on texts (Fairclough, 1995), considering hegemony and power relations. Furthermore, CDA allows the interpretation of the ideological meaning of texts, and how individuals interpret texts in relation to social structures, social practices, morality, and social relations (Fairclough, 2003), in this case the relationship between migration and modern slavery. Amer and Howarth (2018) used CDA to examine representations of White British Muslims in national UK and Muslim UK newspapers. They established a difference between national and Muslim newspapers, where White British Muslims were a threat due to their position as ‘White British’. Also, the inter‐relatedness of social representations and identity can allow for the exploration of the impact of media representations on identity.

Fairclough's (2010) CDA was utilized in analysing the newspaper articles. From the content analysis, the 5 highest scoring extracts for each class were selected, and the newspaper articles were then located. The social harm was identified, and then key features from the newspaper articles were identified. The structure and style of article were noted. The use of different ‘voices’ was analysed and how they were used to construct the social harm and obstacles to overcoming the harm. Based on principles of Foucault (1972, 1977, 1982, 1990), specifically notions of power and ‘dividing practices’ whereby specific groups are deemed as ‘social problems’ through knowledge that shapes social practices. The analysis will take a social constructionist approach that the discourses serve a purpose of maintaining power through the divisions reproduced according to race and nationality. Furthermore, discourses will be interpretated based on Van Dijk's (1993) work on race, politics and discourse, examining the political purpose of the discourses and how they reproduce specific narratives of race for political gain. Inclusions and omissions were also noted, as newspaper reports tend to be racialised, so the reference (or lack of) was part of the analytic process. Based on Moscovici (2000), the potential consequences were highlighted.

## RESULTS

### Content analysis

**FIGURE 2:**
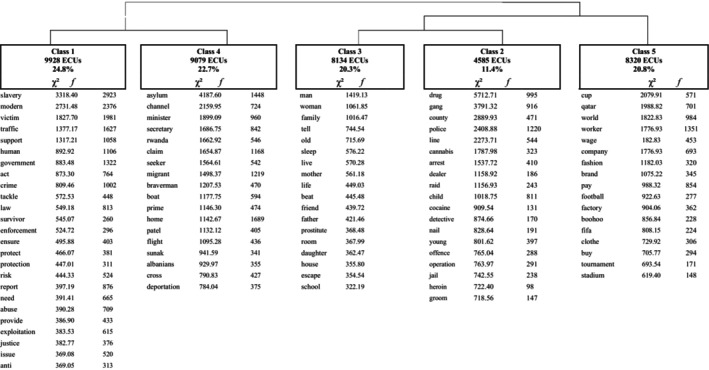
Descendant hierarchical classification (DHC) of 2672 newspapers articles.

The *corpus* analysed contained 2672 newspapers articles published between 2013 and 2022. The DHC analysed 42,521 ST, of which, 36% were utilized to form five classes. Figure 2 shows the five classes, the relationship between classes, the most frequent words and chi‐squares. Class 1 grouped 24.8% of the text segments and was related to Class 4, which grouped 22.7% of the text segments. Classes 2, 3 and 5 were related to each other and grouped, respectively, 11.4%, 20.3% and 20.8% of the text segments. Analysis of the most frequently used words and typical extracts indicated that classes 1 and 4 reflected discourses about the government's role in tackling modern slavery and how the government was treating migrants crossing the channel. The fact these two classes were inter‐related in the analysis, highlight the fuzzy boundaries between modern slavery and immigration. The second set of inter‐related classes (2, 3 and 5) grouped articles that discussed specific cases in relation to police actions and court rulings, as well as articles reporting the world cup in Qatar. Since classes 1 and 4 better addressed the research question, the typical extracts and articles in these classes were used for subsequent analysis.

### CDA

Two distinct yet inter‐related themes emerged from the content analysis that relate to the double standard that trafficked people face when being forced to migrate to the UK. The first theme is ‘making claims’ which focuses on modern slavery as criminality, focusing on the wrong‐doing of minority groups, as well as language around the veracity of claims of refugee and victimhood by migrants in the UK. Where modern slavery is accepted, how this is negotiated as a British problem is discussed. The political and social implications of challenging the authenticity of trafficking claims will be considered. The second theme is ‘Leading the way in tackling modern slavery’. This theme identifies historically the position of the UK as fighting against slavery, and now taking up the mantel of fighting modern slavery. This theme examines how the media construct the government as moral gatekeepers, representing the anti‐slavery stance as part of British identity. These themes combined highlight how discourses are used to justify the government's policies around modern slavery and migration.

### Themes 1: Making claims

This theme explores the constructed veracity of victims of modern slavery in the backdrop of discourses of immigration in the UK. Here, rather than gendering and racializing modern slavery, the theme focuses on how political and media sources use discourses to delegitimise victims of modern slavery and trafficking. By drawing upon specific racial groups, allows for the media to question modern slavery claimants, with the headline ‘Tory MP: Let's deny all Albanian claims’ (Daily Mail, November 29th 2022). The force of facts (Van Dijk, 1993) is used here, with the article drawing upon data to construct Albanians as the majority of asylum claimants. The use of this example, allows the article to generalize their claims against Albanians wider.

Mr. Davis said: ‘The longer we allow this to go on, the more people will be able to claim family ties in the UK or find loopholes in our human trafficking and modern slavery laws’.

The shift here is from Albania and asylum seeking, to ‘people’ and different legislation. The use of politicians here legitimizes the claims that the migrants in question are acting illegally. The authenticity of modern slavery claims by migrants comes into question with the following heading in The Telegraph (November 28th, 2022).

MPs urge action on ‘bogus’ asylum seekers; 50 Conservatives urge the PM to close legal loopholes to deter further Channel crossings.

The article is based on a letter with 50 signatories, including members of the cabinet and the 1922 committee, which gives a sense of political power to the letter.

They argue that ‘people claiming they have been unwilling victims of human trafficking or modern slavery’ should be returned to ‘the villages from which they came’. ‘If they have really been taken against their will, then they could not reasonably object to being returned to their own homes,’ the letter explained.

In this extract which consists of a direct quote from the letter uses discourses of deviance and criminality, but also of racial nationalism and colonialism. The ‘claimants’ are constructed as tribal (living in villages) and ‘fake’, seeking a better life in the UK. The quote here simplifies the issue and uses a sense of straw‐man logic (could not reasonably object). There is this construction of British superiority and colonialism, with the assumption of village dwelling migrants as opposed to more sophisticated Brits. The reference to 50 signatories shows a strength of unity, that this is collective action and based on democracy. Van Dijk (1993) argued that refugees are often delegitimised using economic refugees, equating to fakery. The article in The Telegraph uses such political discourses.

NCA chiefs said a ‘significant number’ of the Albanians in the UK had entered illegally to work in the ‘grey’ market or for organized criminal drug gangs and were sending back ‘hundreds of millions of pounds’ a year to Albania. They said there was evidence from Albania that migrants were told before they left for the UK that if they chose to join crime gangs and were caught, they should claim to be victims of modern slavery in order to avoid deportation and remain in the UK.

The use of the National Crime Agency here gives a sense of authority to the claims. The migrants are homogenized and criminalized, who use British legislation to prevent deportation and to continue to profit in the UK. The use of ‘choice’ is significant, as the Modern Slavery Act 2015 states that victims of modern slavery who are forced to commit crimes should not be criminalized. Here the construction is of culpability, that there is a choice to commit criminal acts. The social harm here is the presence of the migrants, and the Modern Slavery Act is the obstacle that prevents the government from dealing with this effectively.

In another article (The Independent, October 4th, 2022), Suella Braverman is the political voice, constructing legislation of protection (European Rights Convention and the Modern Slavery Act) as not fit for purpose and a source of exploitation of the UK.

Ms. Braverman told the conference in Birmingham the law ‘simply isn't working’ and legislation was being ‘abused’ by people smugglers, people making ‘multiple, meritless and last‐minute claims’ and – taking aim at lawyers – by ‘specialist small’‐ boat chasing law firms.

Here political discourses of negative presentation of others (Van Dijk, 1993), by using depictions of manipulation and criminality. She is also invoking a moral comparison with lawyers, drawing upon discourses like ‘ambulance chasers’, making immoral claims for personal, financial gains. Here migrants are criminalized and identified as the social harm, and the legal system is the obstacle. She denies any claim of racism.

Setting out her intentions to ensure UK immigration policy is not ‘derailed’ by modern slavery laws, the Human Rights Act or the European Court […] Ms. Braverman insisted it is not ‘racist’ to ‘want to control borders’, or ‘bigoted to say that we have too many asylum seekers who are abusing the system.’

‘It is not xenophobic to say that mass and rapid migration places pressure on housing, public services and community relations. I reject the Left's argument that it is hypocritical for someone from an ethnic minority to tell these truths’ she added.

Here the political discourses drawn upon are denial of racism, as well as ‘apparent sympathy’ (Van Dijk, 1993), she is positioning her call as beneficial for the UK and its systems. She is also transferring racism to the ‘Left’, by constructing the othered, ‘Left’ as highlighting her ethnicity. She is shifting the blame and using discourses of force of facts, she is ‘telling the truth’. This blame shifting is apparent in an article in the Daily Mail (December 31st 2022), with the headline ‘Arise, Sir Border Farce; New Year Honours Honoured, the top civil servant blamed for failing to sort out the migrant crisis’. The article invokes a disdain for elitism, ‘THE Home Office's top mandarin receives a knighthood today despite being blamed for failing to tackle the immigration crisis and broken asylum system’. The anti‐elitism is further amplified with claims that *Permanent Secretary Matthew Rycroft is accused of thwarting ministers' attempts to* *stop illegal Channel crossings and undermining the plan to deport migrants to* *Rwanda. He is one of several senior civil servants caught up in controversy to be* *given gongs in the New Year Honours List, sparking a row over ‘rewards for failure’* *[…]*.

‘*Handing him a knighthood is not only rewarding failure, it's rewarding someone* *who blocked the elected Government's attempts to secure Britain's border*’ [*…*].

*Sir Matthew is said to have opposed a series of tough measures to deter Channel* *crossings, including closing a loophole in modern slavery laws that has been* *exploited by Albanians*.

Here the article is invoking images of the incompetent, elite as undermining democracy and legislation. Again, the undemocratic elite are barriers to dealing with what is constructed as the harm, which is the homogenized migrant. The UK government constructed through the media is one of morality and fairness. The discourses focus on the superiority of the government in terms of commitment and success in doing the right thing. Modern slavery is described using discourses of barbarism and immorality, with little focus on the victims. Instead of differentiating migrants, the media constructs the migrant as causing criminal acts and those who protect them are obstacles to preventing the social harm they cause.

### Theme 2: Leading the way in tackling modern slavery

This theme examines how modern slavery is constructed as a social problem from a national and international perspective. Here discourses of moral nationalism, duty and pride will be set against the ethnic othering to construct a sense that the UK is standing against modern slavery, due to narratives of Great Britain historically leading the way in abolishing slavery. The extracts from The Independent (19/04/2018) takes the position of the UK government taking an international stance against modern slavery, to root out human trafficking and child exploitation in some of the poorer countries in the Commonwealth […] for an end to ‘one of the greatest injustices of our times’ as she announced a package of measures to stamp out forced labour and trafficking affecting more than 40 million people across the world.

Here morality and criminality are used in juxtaposition to position the UK government as morally superior. The extract also uses distancing by placing modern slavery as the plight of ‘poorer’ countries, using geographical, economic and moral distancing from modern slavery. The article is invoking a sense of national pride and justice, and the ‘strong‐arm’ of the law, as the moral saviour of the commonwealth, which is reminiscent of colonial discourses. The use of a direct quote from Penny Mordaunt reinforces the representation as a problem external of the UK.

The UK and the Commonwealth are stepping up to fight one of the greatest injustices of our time—the trafficking and exploitation of vulnerable people by predators. UK aid is helping to stamp out these evil practices, by smashing the traffickers' exploitative business model, helping to punish the perpetrators and supporting vulnerable people and victims—who are all too often women and children—to rebuild their lives so they do not fall back into a cycle of abuse.

Here there is a sense of unity with the UK leading the fight. The use of ‘stepping up’ invokes a sense of leadership but also one of not avoiding responsibility. The use of visceral language such as ‘smash’ serves to intensify the moral power of the UK, and the cultural justices of the UK for children and women. The article goes on to state that the UK is ‘leading the world’ with the ‘groundbreaking’ Modern Slavery Act 2015. The article draws upon a sense of moral superiority of the UK government and the UK, invoking a sense of national pride and duty on the world stage to lead the way once again in tackling slavery. The article positions the UK as ‘paternalistic’, protecting the poorer nations and their most vulnerable citizens. By using geographic distancing, there is no direct responsibility for the UK government here, so the involvement is a moral one. This article suggests that, even though the UK are supporting the fight against modern slavery and human trafficking, this is not a ‘British’ problem.

This can potentially be problematic when instances of modern slavery and trafficking are identified in the UK. An instance of a raid by the police was reported in multiple news outlets, and in these the law and order of the UK are positioned as in direct opposition to modern slavery. In The Mirror (28 December, 2016) and The Independent (28 December, 2016) both state the number of arrests. In the articles they focus on those who commit the offence rather than the victims. Moral distancing was used in both articles. This operation sends a strong message to those employers who ruthlessly seek to exploit vulnerable people and wilfully abuse our immigration laws. Modern slavery is a barbaric crime which destroys the lives of some of the most vulnerable in our society (The Independent, 28th December 2016).

Here there are two groups of ‘actors’, the police who are not only upholding the law but serving a vulnerable society. This is contrasted with the assailants, described as ‘barbaric’, which in the context of how they are described racializes the barbarism. Both newspaper article uses the same description, ‘The majority of the 97 people arrested were Vietnamese nationals, but the number also included suspected immigration offenders from Mongolia, Ghana, China, Nigeria, Pakistan and India’ (The Independent, 28th December 2016). This is reminiscent of Billig's (1995) observations of order of nations when examining political discourse, where there are certain ‘taken for granted’ issues that relate to race, taking the racialized perspective that specific nations are more deviant and/or criminal. Here the use of language such as ‘barbarism’, alongside the force of facts (Van Dijk, 1993) whereby data relating to nationality is used constructing the criminals racially and othering them. It was noted that there were 14 victims, however the nationality of the victims was not stated in the articles. This omission is informative, as the narrative here is one of criminality rather than the victims of modern slavery and trafficking.

The Mirror's article (28th December 2016) further amplifies the superiority of the Government which is described as taking ‘world‐leading action to tackle it by introducing the Modern Slavery Act, giving law enforcement agencies the tools they need and increasing support and protecting victims’. Discourses of justice and morality are used in The Telegraph (November 18th, 2014), with the police having “ground‐breaking’ new crime‐fighting powers’. The article focuses predominantly on discourses of law and order and the new powers of the law, describing the event before contrasting this with the details of the case.

*Five people were charged last week for their part in the trafficking ring, which, allegedly tricked a pregnant Eastern European woman to travel to the UK before selling her to an Asian family and forcing her into a sham marriage*.

*The Slovakian victim claimed she had entered the country on the belief that she would be meeting her sister here*.

*Police have said that the going rate for a sham marriage with a ‘standard’ female was £3000*.

*It rose to between £10,000 and £15,000 for a pregnant women because it practically guarantees illegal immigrants the leave to remain in the UK*.

There is a level of detail here to construct the necessity of these new powers and to place the law and order of the British system as superior to the depravity of traffickers. Including the story of the victim ‘anchors’ the concept of the trafficker here. The inclusion of ethnicity serves to distance modern slavery from UK nationals and allows for moral comparisons to be drawn (Moscovici, 2000), but also further anchors the crime, by drawing upon already existing representations of arranged marriages and the commodification of women in human trafficking. The force of facts in terms of the ‘value’ of the woman is used by identifying the monetary value of the women. traffickers. Furthermore, it indicates that the traffickers are aware of ‘loopholes’ in the law that can be manipulated. The success of the moral, just and legal stance of the UK and law enforcers is highlighted in these articles. Here the issue of trafficking as a social harm is located as being the fault of criminal migrants, while the British legal system is the solution to the social harm.

Even when the system fails, it is the ethnic others that are to blame. For instance, The Telegraph (November 2nd, 2022) reported on the failing of modern slavery law. The article draws upon the words of Theresa May.

‘Modern slavery’, she said, was a ‘barbaric evil’ that must be defeated. Women were being trafficked to the UK and forced into prostitution while children were ‘being made to pickpocket on the streets and steal from cash machines’.

The distancing here is subtle, using ‘barbaric’, and the use of ‘to the UK’ positions modern slavery as an issue that is not native to the UK. The victims are constructed as vulnerable based on age and gender. In sharp contrast, 7 years on, critics suggest the legislation is being manipulated and exploited by Channel‐crossing migrants—and specifically Albanian men—who have come to the UK illegally and then invoke the act to prevent their subsequent deportation.

These two extracts give two different perspectives of the Modern Slavery Act 2015, the first is the UK Government's perspective of morality, the second is the othering of migrants as exploitative. This constructs two sets of ‘actors’ in modern slavery, the morally superior UK legal and political system, and the deviant, manipulative, young, male migrant. This invokes a sense of nationalist morality and moral comparisons to be drawn. The article also negates British citizens of any responsibility by depicting modern slavery as hidden.

‘It is hard to comprehend that such sickening and inhuman crimes are lurking in the shadows of our country…From nail bars and car washes to sheds and rundown caravans, people are enduring experiences that are simply horrifying in their inhumanity.’

This quote from Theresa May serves to depict modern slavery as alien from the everyday lives of UK citizens, the ‘hidden in plain sight’ discourse here means that ordinary citizens are not accountable. Theresa May here is a source of authority and expertise, to distance citizens from crimes associated with modern slavery. The article uses statistics (the force of facts) to problematise the issue ‘almost 40,000 people have illegally crossed the Channel […] In the whole of 2021, 28,526 people […] to date 12,000 Albanians had made the crossing this year, up from 800 in 2021[…] Albanians had become the top nationality claiming to be trafficking victims’. The use of numbers here amplifies the ‘problem’ and then it is framed around the Modern Slavery Act, stating that the legislation is being ‘being exploited by Albanians to boost their chance of asylum’, reinforcing the idea that specific, deviant individuals are wrongly seeking asylum This racializing and criminalisation of modern slavery serves to delegitimise claims for asylum while maintaining the idea that the UK government are committed to tackling modern slavery.

This theme manages to serve two purposes, the first is to provide a positive self‐presentation of political figures and the negative other presentation (Van Dijk, 1993). This theme constructs a double bind for migrants and victims of modern slavery. The focus on criminalizing and the othering of ethnic groups serves to marginalize and delegitimise them. The focus on newspaper articles is on the criminality and barbarism of traffickers, and the exploitation of the ‘just, fair and moral’ UK systems. The use of personal stories aims to gender victims of modern slavery, and focus on the vulnerability of victims, as pregnant, women, children. Whereas, those exploit the system are gendered, with the emphasis on the 10,000 men from Albania (The Telegraph, November 2nd, 2022). The morality and justice of the UK is emphasized, drawing upon racist ideologies of the impact of migration on our social order, either through the presence of modern slavery and trafficking, or the exploitation of the UK's legal system. This forms to maintain the ‘systems of differentiation’ (Foucault, 1977) where the migrant is othered and criminalized.

Combined, these two themes show the double bind for victims of modern slavery. According to Van Dijk (Van Dijk, 1993; 1998, 2015) the media and politicians are part of elite power constructs that work together to maintain power. On the one hand, political and media discourses position the UK systems as groundbreaking in combatting modern slavery, invoking a sense of pride in the UK for tackling modern slavery on the international stage. On the other hand, there is a distancing from modern slavery and victims, they are not in the UK, and if they are they are the victims of ethnic groups. They are invisible, negating UK citizens of any responsibility for modern slavery in the UK. Those who claim to be victims of modern slavery are criminalized, and constructed as deviants who are abusing the system. Such social representations serve to maintain a sense of moral superiority for the political systems and power structures, and the readers of the newspapers, as they can make moral comparisons that relieve them of any culpability. However, these social representations serve to silence victims, and to create fear in reporting the crimes committed against them, for fear of deportation and criminalisation.

## DISCUSSION

This study investigated emerging discourses around modern slavery and migration in UK newspaper media between the years of 2013 and 2022. Mass media communication can be considered a distinct social reality, at the same time it creates and propagates social representations (Rochira et al., 2020). SRT provided a framework to understand consensual universes expressed in discourses and practices. Through content analysis we identified two inter‐related themes, ‘Leading the way in tackling slavery’ and ‘Making claims’, which were analysed in depth for the most representative articles within each theme using CDA. Overall, our findings evidenced the double standards regarding the status of individuals who have been trafficked.

### Summary of findings

The first theme, ‘making claims’, identifies labelling discourses of minority groups in terms of wrong‐doing and criminality, and discourses questioning the veracity of victimhood. From the perspective of social justice for modern slavery victims, these social representations serve to silence victims, and to create fear in reporting the crimes committed against them, for fear of deportation and criminalisation. Moreover, the media discourse constructs individuals' knowledge about modern slavery in parallel with the immigration debate: the unfamiliar (victim of modern slavery) is anchored to pre‐existing schemes (migrants), corroborating SRT (Moscovici, 2000). Such social representations serve to shape UK citizens social identity, to maintain a sense of moral superiority for the political systems and power structures, and to relieve UK citizens from any feelings of culpability. It is important to note that although immigration issues were salient within this them, our search did not include any terms related to immigration. This highlights the ability of mass communication to shape people's perceptions of issues that should, in practice, remain separate.

The second theme, ‘Leading the way in tackling modern slavery’, identifies a narrative of the UK as morally superior in its stance against (modern) slavery. Such narrative perpetuates colonial and paternalistic discourses, as identified in the article in The Independent, which placed the UK as the saviour of the Commonwealth, especially of poorer countries. Findings evidenced how the media construct the government as moral gatekeepers, representing the anti‐slavery stance as part of British identity. Different devices are used to ‘other’ perpetrators of modern slavery and shape their social representation, such as evidencing ethnicity or cultural customs such as arranged marriages. Through this narrative, UK nationals can distance modern slavery from themselves, negate any responsibility and can draw moral comparisons (Moscovici, 2000). This theme also serves to problematise migrants and asylum seekers. This serves the purpose of positioning the government as acting morally and legally and any perceptions that contradict this are due to the ‘false’ claims.

### Policy implications

Current UK legislation treats modern slavery and immigration, especially asylum seeking, as completely separate issues. The support available for individuals is also differs depending on their status. While the asylum legislation and process provide housing and a durable solution in terms of right to remain in the country, victims of modern slavery do not have the same access to support and legal status. Thus, the lack of status of modern slavery victims increases their vulnerability through lack of access to services and rights. However, the narrative of asylum seekers, illegal immigration and modern slavery are conflated, with the now Labour government claiming their actions are to ‘smash the gangs’ (Piper, 2023). The current study argues that the legal distinction between modern slavery and immigration is not replicated in media discourses and political debates. This causes confusion and dissatisfaction, which reached a point where a weekend of rioting blighted the UK where communities and hotels were targeted, but the issues unclear (De Simone, 2024). Given that both immigration and modern slavery cases are handled by the Home Office, there is a need for policy changes to create a more straightforward process for individuals whose circumstances fall within the blurred lines of modern slavery and immigration. There is also a need for a clearer message as to how different groups are defined in society and how this is conveyed in the media.

### Limitations and future directions

Media analysis is a well stablished method in social psychology, but it is not exempt from limitations. In this study, the use of Iramuteq and content analysis offered agility and rigour to process a large amount of textual data (Ramos et al., 2018; Sarrica et al., 2016), however, the strategy to analyse the data into a single corpus meant that a large proportion of the corpus was not accounted in the lemmatisation process. Although concerns about the reliability of the lemmatisation process could be raised, previous investigations have demonstrated that Iramuteq provides the same quality lemmatisation as other commercial software (Sarrica et al., 2016).

Recent events in the UK suggest that there is a need to examine how discourses of modern slavery and immigration have evolved. This study offered a snap‐shot of two key themes that serve political policy. However, recent events have highlighted that the disparity between the reality of migration in the UK and modern slavery, and the perceptions of the public have reached a dangerous point. Therefore, it is proposed that future research would maintain the mixed methods approach, but offer a longitudinal perspective. This would allow the researchers to identify key political and social events (e.g. Brexit, Black Lives Matter protests) and the reproduction of discourses, to examine how these have developed over time to the tipping point reached now in the UK.

In summary, the current paper contributes to understanding how discourse about modern slavery in newspaper media in the UK shapes people's view and social representations. In our findings, two themes evidenced a narrative that reinforces power dynamics by positioning the UK as a role model in tackling modern slavery at the same time it reinforces anti‐immigration narratives. Moreover, the findings highlighted that modern slavery is politically constructed. The influence of mass media on individuals' social representations is inevitable, thus, our investigation found the call for action to combat gendered, racialised and paternalistic narratives in UK newspapers.

## AUTHOR CONTRIBUTIONS

**Melanie Haughton:** Conceptualization; methodology; writing – original draft; writing – review and editing; investigation; formal analysis; project administration; resources. **Katia C. Vione:** Conceptualization; investigation; writing – original draft; writing – review and editing; methodology; software; formal analysis; resources. **Zoe Hughes:** Resources; writing – review and editing; validation.

## CONFLICT OF INTEREST STATEMENT

There are no conflicts of interest.

## Data Availability

The data that support the findings of this study are available from the corresponding author upon reasonable request.

## References

[bjso12824-bib-0001] Amer, A. , & Howarth, C. (2018). Constructing and contesting threat: Representations of white British Muslims across British national and Muslim newspapers. European Journal of Social Psychology, 48(5), 614–628.

[bjso12824-bib-0002] Anti‐Slavery International . (2024). What is modern slavery? Anti‐Slavery International. https://www.antislavery.org/slavery‐today/modern‐slavery/#:~:text=At%20Anti‐Slavery%20International%2C%20we%20define%20modern%20slavery%20as,to%20human%20trafficking%2C%20forced%20labour%20and%20debt%20bondage

[bjso12824-bib-0003] Arnell, P. , Lewis, O. , Kalocsányiová, E. , & Forrester, A. (2023). The UK's illegal migration bill: Human rights violated. Medicine, Science, and the Law, 63(4), 267–269. 10.1177/00258024231186736 37487204

[bjso12824-bib-0004] Augoustinos, M. (2001). Social categorization: Towards theoretical integration. In K. Deaux & G. Philogène (Eds.), Representations of the social (pp. 201–216). Blackwell Publishers.

[bjso12824-bib-0005] Balch, A. (2023). 19. How the UK's new immigration law will put more people at risk of modern slavery. https://theconversation.com/how‐the‐uks‐new‐immigration‐law‐will‐put‐more‐people‐at‐risk‐of‐modern‐slavery‐209746

[bjso12824-bib-0006] Billig, M. (1995). Banal Nationalism. Sage publications.

[bjso12824-bib-0007] Birks, J. , & Gardner, A. (2019). Introducing the slave next door. Anti‐Trafficking Review, 13(1), 66–81. 10.14197/atr.201219135

[bjso12824-bib-0008] Cole, M. (2019). Theresa May, The Hostile Environment and Public Pedagogies of Hate and Threat: The Case for a Future Without Borders.Routledge.

[bjso12824-bib-0009] Dando, C. J. , Walsh, D. , & Brierley, R. (2016). Perceptions of psychological coercion and human trafficking in the west midlands of England: Beginning to know the unknown. PLoS One, 11(5), e0153263. 10.1371/journal.pone.0153263 27149330 PMC4858279

[bjso12824-bib-0010] De Noronha, L. (2019). Deportation, racism and multi‐status Britain: Immigration control and the production of race in the present. Ethnic and Racial Studies, 42(14), 2413–2430. 10.1080/01419870.2019.1585559

[bjso12824-bib-0011] De Simone, D. (2024). August 21. Riots show how the UK's far right has changed.

[bjso12824-bib-0012] Fairclough, N. (1995). Media discourse. Hodder Education.

[bjso12824-bib-0014] Fairclough, N. (2003). Analysing discourse. Routledge.

[bjso12824-bib-0015] Fairclough, N. (2010). Critical discourse analysis (2nd edition). Routledge.

[bjso12824-bib-0016] Foster, P. , & Borrett, A. (2024). UK immigration: Why public opinion is at odds with reality. Financial. Times https://www.ft.com/content/5a00c171‐8194‐4c54‐9ac6‐63ca292522e2

[bjso12824-bib-0017] Foucault, M. (1972). The archaeology of knowledge. Tavistock.

[bjso12824-bib-0018] Foucault, M. (1977). Discipline and punish: The birth of the prison. Allen Lane.

[bjso12824-bib-0019] Foucault, M. (1982). The subject and power. Critical Inquiry, 8(4), 777–795.

[bjso12824-bib-0020] Foucault, M. (1990). Politics philosophy culture: Interviews and other writings 1977–1984. Routledge.

[bjso12824-bib-0021] Fraser, E. (2024). What does the Rwanda plan mean for victims and survivors of human trafficking? Bond. https://www.bond.org.uk/news/2024/03/what‐does‐the‐rwanda‐plan‐mean‐for‐victims‐and‐survivors‐of‐human‐trafficking/

[bjso12824-bib-0022] Free, W. (2024). Global slavery index. Walk Free. https://www.walkfree.org/global‐slavery‐index/

[bjso12824-bib-0023] Hulland, L. (2020). Stolen lives: Human trafficking and slavery in Britain today. Sandstone Press.

[bjso12824-bib-0024] May, T. (2016). Defeating modern slavery: Article by Theresa May. Gov. UK. https://www.gov.uk/government/speeches/defeating‐modern‐slavery‐theresa‐may‐article

[bjso12824-bib-0025] Montalescot, L. , Lamore, K. , Flahault, C. , & Untas, A. (2024). What is the place of interpretation in text analysis? An example using ALCESTE® software. Qualitative Research in Psychology, 21(2), 200–226. 10.1080/14780887.2024.2316624

[bjso12824-bib-0026] Moscovici, S. (2000). Social representations: Explorations in social psychology. New York University Press.

[bjso12824-bib-0027] Mynott, E. (2000). Analysing the creation of apartheid for asylum seekers in the UK. Community, Work & Family, 3(3), 311–331.

[bjso12824-bib-0028] Observatory, M. (2023). How are attitudes to immigration in Britain changing? Migration. Observatory https://natcen.ac.uk/how‐are‐attitudes‐immigration‐britain‐changing#:~:text=The%20general%20picture%20shows%20that%20while%20views%20towards,positive%20views%20towards%20immigration%20since%20the%20survey%20began

[bjso12824-bib-0029] Home Office (2010). Immigration: Home Secretary's speech of 5 November 2010. Home Office. https://www.gov.uk/government/speeches/immigration‐home‐secretarys‐speech‐of‐5‐november‐2010

[bjso12824-bib-0030] Piper, E. (2023). 'Smash the gangs': UK labour leader sets out plans for illegal migration. Reuters. https://www.reuters.com/article/world/uk/smash‐the‐gangs‐uk‐labour‐leader‐sets‐out‐plans‐for‐illegal‐migration‐idUSKBN30K0T6/

[bjso12824-bib-0201] Ramos, M. G. , Rosáriolima, V. M., & Amaralrosa, M. P. (2018). IRAMUTEQ software and discursive textual analysis: Interpretive possibilities. In *World conference on qualitative research* (pp. 58–72). Springer International Publishing.

[bjso12824-bib-0032] Ratinaud, P. (2009). IRAMUTEQ: Interface de R pour les Analyses Multidimensionnelles de Textes et de Questionnaires. http://www.iramuteq.org

[bjso12824-bib-0033] Reinert, M. (1993). Les ‘mondes lexicaux’ et leur ‘logique’ à travers l'analyse statistique d'un corpus de récits de cauchemars. Langage et Societe, 66(1), 5–39. 10.3406/lsoc.1993.2632

[bjso12824-bib-0034] Rochira, A. , Salvatore, S. , Veltri, G. A. , Redd, R. R. , & Lancia, F. (2020). Theory and method for the analysis of social representations. In Theory and method for the analysis of social representations Media and social representations of otherness (pp. 17–38). Psycho‐social‐cultural implications.

[bjso12824-bib-0035] Rodríguez‐López, S. (2018). (De)constructing stereotypes: Media representations, social perceptions, and legal responses to human trafficking. Journal of Human Trafficking, 4(1), 61–72. 10.1080/23322705.2018.1423447

[bjso12824-bib-0036] Sarrica, M. , Mingo, I. , Mazzara, B. , & Leone, G. (2016). The effects of lemmatization on textual analysis conducted with IRaMuTeQ: Results in comparison, JADT2016: 13ème Journées Internacionales d'Analyse Statistique de Données Textuelles.

[bjso12824-bib-0037] Shraer, M. (2019). Modern slavery: What has Theresa May done to tackle it? BBC News https://www.bbc.co.uk/news/uk‐politics‐48593081

[bjso12824-bib-0038] van Dijk, T. (2015). Discourse and racism: Some conclusions of 30 years of research. In A. Capone & J. Mey (Eds.), Interdisciplinary studies in pragmatics, culture and society (pp. 285–295). Springer International.

[bjso12824-bib-0039] Van Dijk, T. A. (1993). Elite discourse and racism. Sage Publications.

[bjso12824-bib-0040] Van Dijk, T. A. (1998). Ideology: A multidisciplinary approach. Sage Publications.

[bjso12824-bib-0041] Warwick University . (2022). Enoch Powell and the 1968 ‘Rivers of blood’ speech. Warwick University. https://warwick.ac.uk/services/library/mrc/studying/docs/racism/powell/

